# New Insights into Understanding Irreversible and Reversible Lithium Storage within SiOC and SiCN Ceramics

**DOI:** 10.3390/nano5010233

**Published:** 2015-02-24

**Authors:** Magdalena Graczyk-Zajac, Lukas Mirko Reinold, Jan Kaspar, Pradeep Vallachira Warriam Sasikumar, Gian-Domenico Soraru, Ralf Riedel

**Affiliations:** 1 Institut für Materialwissenschaft, Technische Universität Darmstadt, Jovanka-Bontschits-Straße 2, 64287 Darmstadt, Germany; E-Mails: reinold@materials.tu-darmstadt.de (L.M.R.); jankaspar2@gmail.com (J.K.); pradeep@materials.tu-darmstadt.de (P.V.W.S.); riedel@materials.tu-darmstadt.de (R.R.); 2 Dipartimento di Ingegneria Industriale, Università di Trento, Via Sommarive 9, 38123 Trento, Italy; E-Mail: soraru@ing.unitn.it

**Keywords:** polymer-derived ceramics, lithium-ion battery, SiOC, SiCN, carbon content

## Abstract

Within this work we define structural properties of the silicon carbonitride (SiCN) and silicon oxycarbide (SiOC) ceramics which determine the reversible and irreversible lithium storage capacities, long cycling stability and define the major differences in the lithium storage in SiCN and SiOC. For both ceramics, we correlate the first cycle lithiation or delithiation capacity and cycling stability with the amount of SiCN/SiOC matrix or free carbon phase, respectively. The first cycle lithiation and delithiation capacities of SiOC materials do not depend on the amount of free carbon, while for SiCN the capacity increases with the amount of carbon to reach a threshold value at ~50% of carbon phase. Replacing oxygen with nitrogen renders the mixed bond Si-tetrahedra unable to sequester lithium. Lithium is more attracted by oxygen in the SiOC network due to the more ionic character of Si-O bonds. This brings about very high initial lithiation capacities, even at low carbon content. If oxygen is replaced by nitrogen, the ceramic network becomes less attractive for lithium ions due to the more covalent character of Si-N bonds and lower electron density on the nitrogen atom. This explains the significant difference in electrochemical behavior which is observed for carbon-poor SiCN and SiOC materials.

## 1. Introduction

Due to increasing energy consumption and environmental aspects, there is a growing interest for new energy related materials. Lithium-ion batteries are the most promising candidates and have received much attention in recent years because of their high energy density. Nevertheless, there is still a need of new electrode materials to meet growing safety, stability and high rate capability requirements. Currently, mostly graphitic materials are used as the anode material in lithium ion batteries due to their low price and high reversibility despite their relatively low capacity (372 mAh·g^−1^), instability during long-time cycling and inadequacy for high power applications. Lithium plating was identified as one of the mechanisms which ends the life of a battery more rapidly due to the formation and growth of lithium dendrites. Metallic lithium is typically absent in a lithium-ion (Li-ion) cell under normal conditions of operation. However, under high current charge rates and/or low temperatures, lithium metal will deposit on the carbon anode in preference to lithium intercalation. To prevent lithium plating at the edges and to avoid anode polarization to a potential close to lithium reduction, the anode electrode has been designed with larger geometry and capacity excess [1,2,3,4,5,6]. The decomposition of the electrolyte and subsequent formation of a film surface layer (solid electrolyte interphase, SEI) causes an increase in the impedance and the consumption of recyclable lithium ions. Moreover, the graphite exfoliation being a consequence of the insertion of solvated Li^+^ in case of insufficient SEI formation brings about accelerated material fatigue. The main reasons of the graphite fatigue are: (i) losses of insertion host and (ii) failure in electronic contact within the electrode material and material with current collector. The addition of various stabilizers, robust electrolyte systems, and temperature treatment are some of the methods that have been adopted to mitigate these aging effects on the electrode [7,8,9,10,11,12,13,14,15,16,17,18].

The studies summarized within this work focus on developing new anodes for lithium ion batteries and understanding the mechanisms of lithium storage. In particular, we investigate silicon-based polymer derived ceramics (PDCs), namely silicon oxycarbide (SiOC) and silicon carbonitride (SiCN), with respect to their electrochemical properties and cycling stability. Silicon-based PDCs are prepared by controlled pyrolysis, in an inert or reactive atmosphere, of preceramic polymers containing Si, H, C, O, N. At lower pyrolysis temperatures (~up to 1100 °C for SiOC and ~up to 1300 °C for SiCN, [19,20,21]), the product after thermal treatment is amorphous. The polymer-derived ceramics route enables designing the chemical and physical properties of the final ceramic material by chemical modification of the preceramic polymer as well as by tuning up the processing route covering the polymer to ceramic transformation [22].

Concerning silicon oxycarbide materials, a set of electrochemical data was published in the middle of the 1990s by the group of Dahn [23,24,25,26,27,28]. However, the above publications mostly address the electrochemical performance of the materials with respect to their elemental composition, without considering the mechanism of lithium storage and lithium transport in these materials. Due to the increased commercial availability of preceramic polymers and the expected industrial application of polymer-derived SiOC based anodes, there is presently a growing interest in ceramic-based electrode materials for Li-ion batteries. As a consequence, questions related to the lithium storage mechanisms within SiOC- and SiCN-based materials have recently been the focus of our research. Ahn *et al.* claim that the mixed bond configuration (tetrahedrally coordinated silicon from SiC_4_ via SiC_3_O, SiC_2_O_2_ and SiCO_3_ to SiO_4_) in SiOC ceramics acts as a major lithiation site [29,30,31,32,33]. Other research groups claim that the Li insertion into carbon-rich SiOC compounds occurs in the form of an adsorption and surface storage within the free carbon phase, similar to the storage of Li-ions in disordered carbons. Host sites are considered the edges of graphene sheets, interstitial and defect sites, micro-pores, graphite nano-crystallites and interfacial adsorption at carbon–crystallite interfaces. The Si–O–C glassy phase, on the contrary, is attributed a minor role in the reversible storage process [34,35,36,37,38,39,40,41,42]. The recent investigation of Fukui *et al.* by means of ^7^Li-NMR (nuclear magnetic resonance) measurements demonstrates that the free carbon phase within these materials is the major hosting site for Li ions [34]. The Li-ion diffusion coefficient (D_Li+_) determined by three different methods, namely potentiostatic intermittent titration technique (PITT), galvanostatic intermittent titration technique (GITT) and electrochemical impedance spectroscopy (EIS) revealed D_Li+_ of about 10^−9^–10^−11^ cm^2^·s^−1^, similar to that reported for disordered carbons (10^−10^–10^−11^ cm^2^·s^−1^) and on average faster than for graphite (10^−9^–10^−13^ cm^2^·s^−1^). This agreement, again, emphasizes the Li-ion uptake in the free carbon of the SiOC microstructure; hence it determines the storing kinetics. Interestingly, the analyzed diffusion coefficient has been found to have less potential compared to disordered carbon and graphite [43].

Though Dahn *et al.* filed a patent related to the use of silazane-derived SiCN ceramics in 1997 which show reversible discharge capacities up to 560 mAh·g^−1^ [44], much less research has been done since that time on the application of these materials in lithium-ion batteries in comparison to SiOC. Pure polymer-derived SiCN materials obtained from polysilylethylendiamine have been investigated by Su *et al.* [45] and Feng [46]. The work of Su *et al.* showed a first discharge cycle capacity of 456 mAh g^−1^ but the material suffered from strong fading with cycling. This problem of capacity fading was solved by Feng by applying an additional heat treatment to the polymer-derived SiCN material, however relatively low capacities of about 300 mAh g^−1^ were achieved. Promising electrochemical results with regard to the capacity and stability of SiCN derived from high-carbon containing polysilylcarbodiimides have been reported by Kaspar *et al.* [47] and Graczyk-Zajac *et al.* [48]. Within the work of Reinold *et al.* [49], the excellent performance of carbon-rich SiCN materials has been demonstrated, including the discussion of the influence of the molecular polymer structure on the resulting ceramic microstructure and in consequence on the electrochemical performance of the material. Solid state NMR studies on SiCN ceramics clearly identify carbon as the main lithium storage site [50], which is in agreement with the work of Fukui *et al.* on SiOC-based materials [34]. It is also shown that composite anode materials comprised of SiCN/graphite [51] and SiCN/silicon [52] exhibit enhanced electrochemical properties as compared to pure graphite and silicon, respectively. Composite materials based on SiCN/hard carbons [53] demonstrate excellent stability with respect to high current charging/discharging performance.

Within this review, by combining the experimental results of numerous research works, we analyze which structural properties of the silicon carbonitride (SiCN) and silicon oxycarbide (SiOC) ceramics determine the reversible and irreversible lithium storage capacities, cycling stability and define the major differences in the lithium storage between SiCN and SiOC ceramic materials. Replacing oxygen with nitrogen renders the mixed bond Si-tetrahedra unable to sequester lithium. This explains why a significant difference in electrochemical behavior of carbon-poor SiCN and SiOC materials is found. For carbon-rich ceramics, the free carbon phase plays a dominant role bringing about high cycling stability and high reversible capacities but also leads to significant first cycle irreversible capacities due to lithium capturing in some pores and voids between carbon layers.

## 2. Results and Discussion

As the high carbon content of the aforementioned ceramic systems seems to play an important role in the reversible storage of Li-ions, our work focused on the development of carbon-rich Si-based polymer derived SiCN and SiOC anode materials for lithium-ion batteries. The following synthesis strategies were applied: (i) using phenyl-rich pre-ceramic Si-polymers as starting materials [19,38,46,48,54]; (ii) addition of carbon or carbon precursor to Si-based polymers [51,53,55] and (iii) chemical modification of pre-ceramic Si-polymers [39,42,53,56,57]. In the last few years, among the various chemical compositions of SiCN and SiOC compounds, stoichiometries with an exceptionally high content of carbon (>50 wt%) were further considered as perspective anode material in terms of high gravimetric capacity, rate capability and reliable cycling stability. The microstructure of carbon rich SiCN and SiOC is presented and discussed elsewhere [22,58,59,60,61,62,63,64]. Here, we present an overview of the electrochemical properties of various SiOC and SiCN based materials with respect to the amount of free carbon phase and the microstructure. Moreover, we evaluate what kind of microstructural properties of the Si(O,N)C ceramic determine the reversible and irreversible capacities and long cycling stability and we identify the major differences in the lithium storage sites within SiCN and SiOC materials.

**Figure 1:**
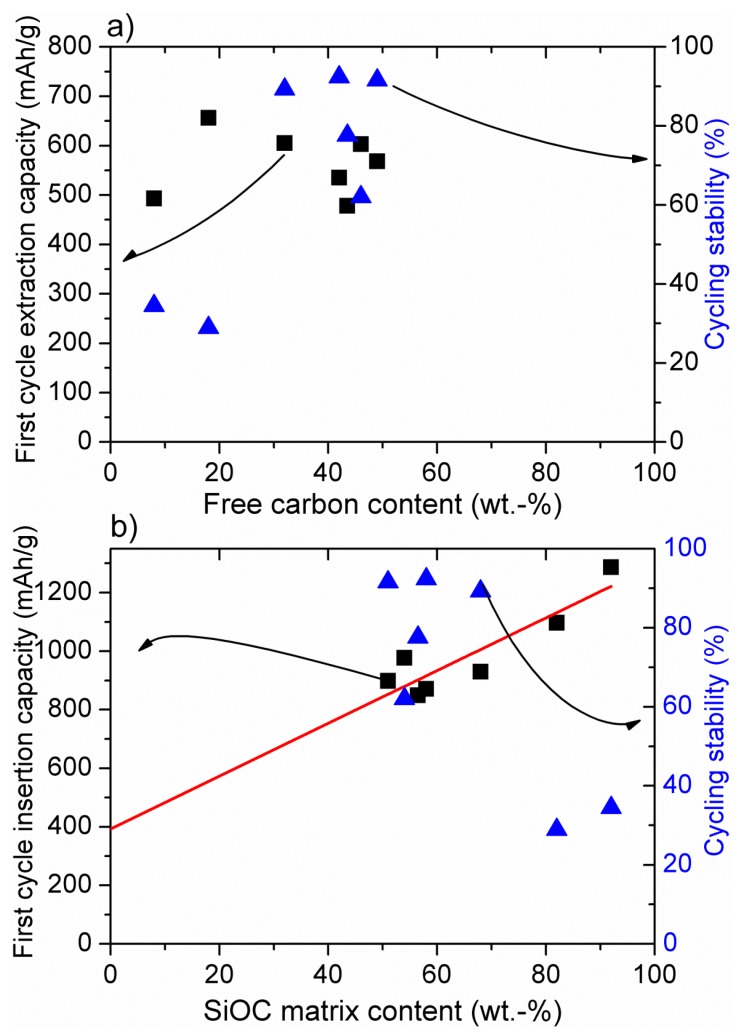
Dependence of the insertion and extraction capacity of SiOC-derived materials on the amount of free carbon (**a**) and SiOC matrix (**b**). Cycling stability defined as the ratio of the extraction capacity after prolonged cycling (<100 cycles) to the first extraction capacity. Experimental data for samples pyrolysed at 1000–1100 °C from [54,56,57].

**Figure 2:**
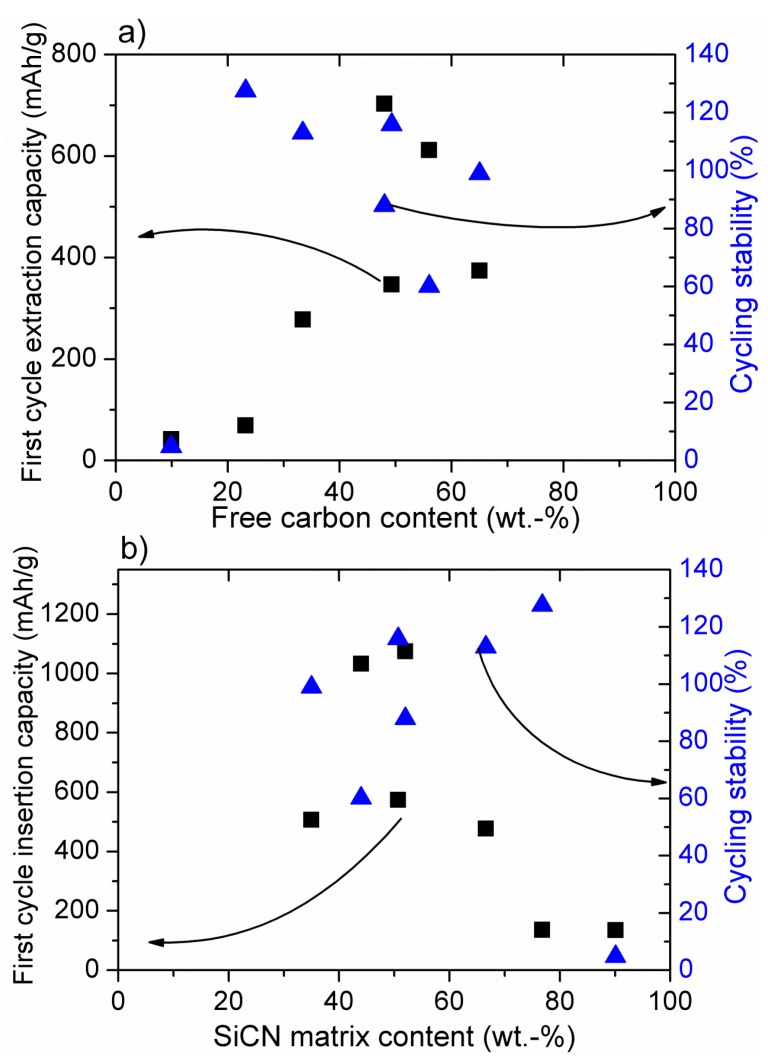
Dependence of the insertion and extraction capacity of SiCN-derived materials on the amount of free carbon (**a**) and SiOC matrix (**b**). Cycling stability defined as the ratio of the extraction capacity after prolonged cycling (>100 cycles) to the first extraction capacity. Experimental data for samples pyrolysed at 1000–1100 °C from [49,51,53,56].

Figure 1a and Figure 2a present the dependence of the first cycle delithiation (extraction) capacity and cycling stability (The cycling stability has been calculated as the ratio of the delithiation capacity after prolonged cycling (>100 cycles) to the first extraction capacity), both registered with low currents (C/10–C/20). The same current was always applied for the charge (C) and for the discharge (D) processes with C/20 = 18 mAh·g^−1^.) from the amount of free carbon phase within SiOC (a) and SiCN (b), while in Figure 1b and Figure 2b the first cycle lithiation (insertion) capacity and stability are plotted as a function of the amount of SiOC (a) and SiCN (b) ceramic matrix (For SiOC materials the amount of free carbon and ceramic phase has been calculated according to the general formula consisting of a stoichiometric silicon oxycarbide network, SiC*_x_*O_2_(_1 − *x*_) and a C_free_ phase [65]). In SiCN it is assumed that oxygen is bonded to silicon as SiO_2_, nitrogen and silicon from Si_3_N_4_ and the remaining Si is bonded to C in the form of SiC. The excess carbon is assumed to exist as free carbon. (%ceramic + %free carbon = 100%). It is obvious that for SiOC delithiation, the capacity does not depend on the amount of free carbon, while for SiCN materials the capacity increases with the amount of carbon to reach a certain threshold value at about 50% of carbon phase [49,56]. The cycling stability of low carbon materials is, however, low for both ceramics.

The data reported in Figure 1b unveil a linear dependence of the insertion capacity from the amount of silicon oxycarbide phase, which is in good agreement with the model proposed by Raj *et al.* [30]. Moreover, the extrapolation of the linear fit down to zero, which means a sample of pure free carbon, indicates a capacity of 400 mAh·g^−1^ and is in agreement with the theoretical capacity of graphite (372 mAh·g^−1^). Interestingly, the insertion capacity of pure SiC*_x_*O_2(1 − *x*)_ phase, which can be obtained by extrapolating the linear fit up to 100% amounts 1300 mAh·g^−1^. However, according to our experience, this capacity cannot be reached practically due to the low electrical conductivity of the materials. According to DFT modelling of the lithium insertion into SiOC materials [66], insertion of Li into amorphous silica (a-SiO_2_) and SiOC containing no free carbon phase is energetically unfavorable due to a large gap between their valence and conduction band.

**Figure 3:**
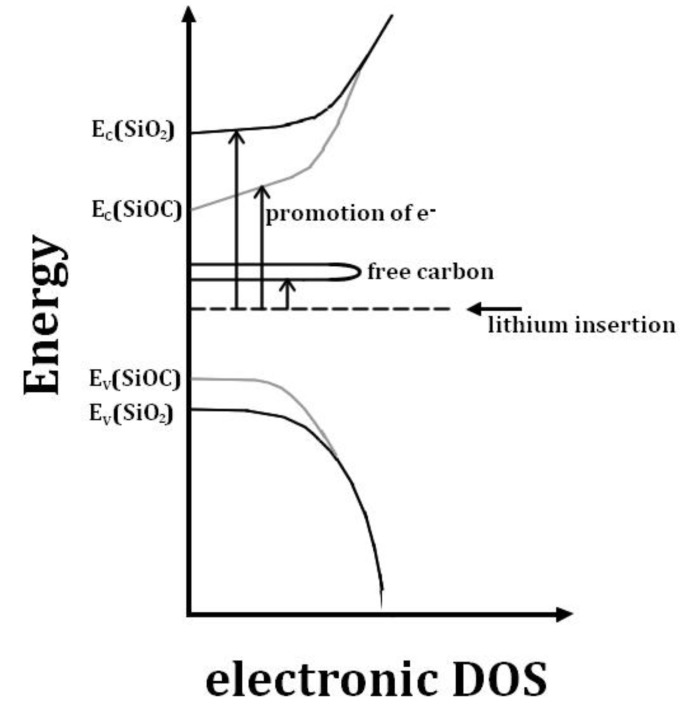
Schematic scheme of the electronic density of states for a-SiO2, SiOC and free carbon according to Reference [66].

In contrast, the presence of a free carbon phase provides low-laying unoccupied states in which electrons can go in, and by consequence significantly decreases the band gap. A graphical illustration of this is given in Figure 3. It shows the electronic density of states (DOS) for a-SiO_2_, SiOC and a free carbon phase according to Reference [66]. A strong bonding of Li in the solid SiOC structure is provided, if the interaction of the lithium cation with the host in the form of Li-O bonds outweighs the promotion energy for the electron. Consequently, on the one hand the free carbon phase facilitates lithium bonded to oxygen sites, leading to irreversible lithium uptake; on the other hand, the segregated carbon provides a major part of the reversible lithium storage capacity [66].

According to Figure 2b, for silicon carbonitride there is no dependence between the first cycle insertion capacity and SiCN matrix amount. The SiCN first lithiation capacity is low for a high amount (>70%) of SiCN matrix. No modelling has been performed on lithium insertion into SiCN materials, yet. However, a qualitative experimental analysis of low carbon SiCN and SiOCN materials has been performed [33]. It has been stated that replacing oxygen with nitrogen renders the mixed bond Si-tetrahedra unable to sequester Li. In SiOC materials, the electronic structure of the mixed bonds can induce a dipole on the Li atom thereby creating a shallow energy well. Mixed bonds that are highly covalent have more localized and “stiff” electron densities, unable to induce such a dipole. Electronegativity values of oxygen and nitrogen amount 3.5 and 3.0, respectively. Therefore, Si–N bonds are more covalent than that of Si–O bonds, which confirms the above argument. This phenomenon corresponds pretty well with our experimental findings. Accordingly, lithium is strongly attracted by oxygen in the SiOC network due to the pronounced ionic character of Si–O bonds, leading to a high electron density on oxygen. This feature brings about high first lithiation capacities, even at low carbon contents. With continuing cycling, lithium is irreversibly captured within the carbon-poor SiOC network leading to low electrochemical stability (see Figure 1a,b). Once oxygen is replaced by nitrogen, the ceramic network is much less attractive for lithium ions due to the pronounced covalent character of Si–N bonds and thus lower electron density on the nitrogen atom. These findings explain the significant difference in the electrochemical behavior of low carbon SiCN and SiOC materials. For carbon-rich SiOC and SiCN ceramics, the free carbon phase starts to play a dominant role bringing about high cycling stability and high reversible capacities, but also leading to significant first cycle irreversible capacities due to lithium capturing, as already discussed [34,50]. From the electrochemical point of view, on the one hand these results suggest that the studied ceramic anodes behave like a composite material comprised of a mixture of pure ceramic SiCN or SiOC matrix and free carbon phase. Nevertheless, it should be noted that the ceramic phase is indispensable to ensure the stability of the free carbon phase within the prolonged cycling. Thus, a certain equilibrium between the free carbon and the ceramic phase has to be maintained in order to achieve the material stability with respect to continuous lithium insertion/extraction. This equilibrium is reached at around a 1:1 weight ratio between free carbon and ceramic phase, but the exact value depends on the particular preceramic polymer and its processing to SiCN or SiOC ceramic.

## 3. Conclusions

In this overview on SiOC and SiCN ceramic negative electrode materials for rechargeable lithium ion batteries, we summarize the experimental results in order to find out the critical material properties influencing their electrochemical performance. For both material types, the calculated amount of SiCN/SiOC matrix or free carbon phase is correlated with the first cycle lithiation or delithiation capacity and cycling stability. It is found that the amount of free carbon phase has no significant impact on the first cycle lithiation and delithiation capacities of SiOC, whereas for SiCN the capacity increases with the amount of carbon until a threshold value is reached at about 50% of carbon phase. The cycling stability of carbon-poor ceramics is very low for SiCN and SiOC. For SiOC, a clear linear dependence of the insertion capacity on the amount of silicon oxycarbide phase is revealed, while for silicon carbonitride there is no dependence between first cycle insertion capacity and SiCN matrix amount. In contrary to the tendency observed for SiOC, the SiCN lithiation capacity is very low for high (>70%) amount of SiCN matrix.

Finally, it is stated that replacing oxygen with nitrogen renders the mixed bond Si-tetrahedra unable to sequester lithium. Lithium is more attracted by oxygen in SiOC network due to more ionic character of Si–O bonds, leading to a high electron density on oxygen. This brings about very high initial lithiation capacities, even at low carbon content. With continuing cycling, lithium is irreversibly captured within the SiOC network bringing low cycling stability. If oxygen is replaced by nitrogen, the ceramic network becomes much less attractive for lithium ions due to the more covalent character of Si-N bonds and lower electron density on the nitrogen atom. It explains why a significant difference in electrochemical behavior of carbon-poor SiCN and SiOC materials is observed. For carbon-rich ceramics, the free carbon phase leads to high cycling stability and high reversible capacity
